# The association of physical activity, body mass index and the blood pressure levels among urban poor youth in Accra, Ghana

**DOI:** 10.1186/s12889-015-1546-3

**Published:** 2015-03-19

**Authors:** Ernest Afrifa–Anane, Charles Agyemang, Samuel Nii Ardey Codjoe, Gbenga Ogedegbe, Ama de-Graft Aikins

**Affiliations:** Regional Institute for Population Studies, University of Ghana, P. O. Box LG 96, Legon, Ghana; Department of Public Health Academic Medical Centre, University of Amsterdam, Amsterdam, The Netherlands; Centre for Healthful Behaviour Change, New York University, School of Medicine, New York, USA

**Keywords:** Blood pressure, Physical activity, Body mass index, Youth, Urban poor

## Abstract

**Background:**

Globally, there is an increasing prevalence of high blood pressure (HBP) among adults and youth. However, the mechanisms of how the risk factors (physical inactivity and obesity) relate with blood pressure (BP) are not well known especially among the urban poor youth in low and middle income countries. Meanwhile childhood and adolescent physical inactivity and obesity, particularly in conditions of poverty, predispose individuals to cardiovascular diseases (CVDs) in later life. The aim of this study was to assess the BP levels and to examine its associations with physical activity (PA) and body mass index (BMI) amongst urban poor youth in Accra, Ghana.

**Methods:**

We studied 201 youth aged 15–24 years in three urban poor communities in Accra, Ghana. Height, weight and BP were measured in all subjects. PA levels were assessed using the Edulink Urban Health and Poverty project questionnaire. Multiple linear regression analysis was used to determine the factors influencing BP levels.

**Results:**

The proportion of pre-hypertension and hypertension among the youth was 32.3% and 4%, respectively. The rates of pre-hypertension (42.0 vs. 24.8) and hypertension (6.8 vs. 1.8) were higher in males than in females. More than three-quarters (84.1%) of the youth were not physically active. Females were more physically inactive compared to the males (94.7% vs. 70.5%). The average BMI was 22.8 kg/m^2^. For overweight (17.7 vs. 6.8) and obesity (13.3 vs. 2.3), females had higher rates than males. BMI was positively related to systolic BP, and significantly associated with systolic BP (β = 1.4, p < 0.000 and β = 0.8, p < 0.000; respectively for male and female youth) compared to diastolic BP. Youth with low PA had raised BP.

**Conclusion:**

The positive association of BMI and BP in the study communities suggests the need for health measures to tackle their increase and related public health consequences. Further studies on BP and other risk factors among the youth of rural populations and other developing countries will be important to stall the rising prevalence and implications for adult morbidity and mortality.

## Background

High blood pressure (HBP) among adolescents affects their health and results in other health problems in later life [1,2]. Yet most studies on blood pressure (BP) have focused on the adult population. Specifically, hypertension is under diagnosed in the youth population [3] since few studies have explored its risk factors in this group [4,5]. Meanwhile childhood and adolescent physical inactivity and obesity as well as certain lifestyle behaviors such as the use of tobacco and alcohol consumption, which are associated with HBP, have increased among the young population [6]. It has been projected that in the next decade, there will be a worldwide increase (15%) in death rates from cardiovascular diseases (CVDs): Africa will record over 20% increase [7]. This will make CVD the most common cause of death compared to communicable diseases and it is projected to affect the younger age population especially in most low and middle-income countries (LMIC) [8]. Most of these deaths will be attributed to HBP [9,10]. Urban poverty is associated with poor living conditions which influence health [11]. In sub-Saharan Africa (SSA), CVD accounts for a considerable proportion of the chronic disease burden [12]. The prevalence of HBP among the aged in West Africa is shown to be 33% in some urban communities [8] however, some studies report a prevalence of between 30-40% for rural communities [13]. Ghana has a high HBP prevalence of 28.7% [14]. A rural–urban study in Ghana suggests a prevalence of 27%, each for both rural men and women and 33.4% and 28.9% for urban men and women respectively [13].

Individual lifestyle behaviors associated with urbanization contribute to the increasing prevalence of HBP in urban areas [4]. Among them are lack of physical activity (PA), alcohol overconsumption, smoking or substance use, unhealthy diets, obesity and psychosocial stress [15]. These factors are now evident and also high among the younger population (15–24) [16]. However, while it is established in most developed countries that PA in adolescents and youth reduces risk of obesity and HBP in later life [17], the association is unclear in most developing countries. For instance in Nigeria, female students in senior secondary schools were more physically inactive compared to their male counterparts (39.9% vs 36.0% respectively; p < 0.05) [18]. In the same study, physical activity level was significantly associated with current educational level and also participants who were inactive were found to have high BMI. Similarly, physical inactivity was found to be accompanied by a high prevalence of overweight and obesity among youth in South Africa [19]. In Cameroon, Sobngwi et al. [20] found among urban and rural respondents 15 years and older, that BP decreased for high energy expenditure.

In Ghana, the overall prevalence of overweight among women aged 15–49 years has increased from 25.5% to 30.5% between 2003 and 2008 [21]. Overweight and obesity which have been attributed to aging are equally now common among adolescents and children due to lifestyle behaviors such as low levels of PA, poor diets and increasing levels of alcohol intake [22]. Childhood obesity increased from 0.5% in 1988 to 1.9% in 1993 and to 5% in 2008 in Ghana. Among the youths aged 15–19 and 20–24 in Ghana, there has been an increase from 7.2% to 9.0% and 15.1% to 16.6%, respectively in the same period [21]. Childhood and adolescent PA has an effect on adult obesity and BP [2,23]. PA reduces risk of obesity, which once established in adolescent and youthful age is hard to reverse [17]. Furthermore, physically active adolescents are at a lower risk of developing other conditions such as type II diabetes in future [24]. Hence BMI and PA are notable factors that relate with BP, and so prevention should begin early in life [25,26]. Also, urban poor areas are predicted to experience increasing burden of HBP and other NCDs as the urban poor context increases several health risks associated with NCDs [11,27]. Rates of NCDs and their risk factors are reported to be on the increase in urban poor areas in Africa [21]. In Ghana, for example, the prevalence rates of HBP have always been higher in urban areas, and the urban rates have increased significantly over the years. Between 2006 and 2007, in the most urbanized Greater Accra Region, hypertension has moved from the 4th to the 2nd place as the primary cause of outpatient morbidity [28]. This study has two main aims: (a) to assess the PA, body mass index (BMI) and BP levels; and (b) to examine the association of PA, BMI and BP by gender of the urban poor youth in Accra, Ghana.

## Methods

### Study areas

We conducted a cross-sectional study among residents of three urban poor areas; James Town and Ussher Town in Ga Mashie and Agbogbloshie between November 25th and December 22nd 2011. James Town and Ussher Town are coastal fishing communities. Both communities are largely indigenous Ga. Residential structures are mostly cement-walled with few places designated for recreational activities. Agbogbloshie on the other hand, is a multi-ethnic migrant community embedded in a major market area. Unlike Ga Mashie, there are mostly closed unplanned residential wooden-walled (kiosk) structures. There are also very few places for recreational activities.

### Study design

The study forms part of the 2nd wave of the cross-sectional ‘Urban Poverty and Health Survey’ (UPHS) conducted by the Regional Institute for Population Studies (RIPS) in December, 2011. RIPS, with support from the Secretariat of the African Caribbean and Pacific Group of States – ACP-EU Cooperation Programme in Higher Education (EDULINK) and IDRC, has established an active research field site in the three study communities. The aim of the UPHS is to examine health, poverty and development indicators in the study areas over time and to provide relevant data to local and regional stakeholders for the development of the communities.

Forty (40) households each were systematically selected from the 29 randomly selected enumeration areas (EAs) of the three localities. With a response rate of 70% only 974 individuals were interviewed after informed consent was obtained. Among them, 201 (62.4% of the proportion of youth) aged 15 to 24 years [29] were sampled. Anthropometric measurements (weight, height, waist and hip circumference) were collected from this cohort. Those who refused to have their anthropometric measures taken had similar demographic characteristics as those sampled; majority had completed Junior High School (JHS) and had a mean age of 19.4 years. Height measurements were obtained using a measuring tape (5 M/16FT measuring tape) in centimeters (cm) after removal of slippers or shoes and a weighing scale (Seca Scale with maximum measurement of 150 kg) provided individuals’ weights in kilograms (kg). PA was self-reported. Respondents were asked about their involvement in an activity in their free time of the past 7 days, whether they had used tobacco in the past 30 days and also if they had taken alcohol in the past 7 days. Similarly, the age, sex, educational attainment, locality of residence and their employment statuses were collected. BP measurements were taken three times using appropriate cuff sizes with a validated electric BP monitor (Microlife® Watch BP®). The measurements were taken within an interval of 2 minutes on either arm of the respondent after ensuring that they were either relaxed or sat appropriately and had not eaten or taken alcohol for the past 30 minutes. Pregnant women were excluded from the study. The average of the three readings was used for the analysis, since there was no difference between the mean BPs with or without the first reading inclusive. The study protocol was approved by the Institutional Review Board (IRB) of the Noguchi Memorial Institute for Medical Research, University of Ghana. As per the IRB approved protocol verbal parental consent was obtained during the household surveys to conduct the study with youth under the age of 16. Once recruited for the study, further consent was obtained from the entire cohort of youth (15 – 24 years) and consent forms were signed or thumb-printed.

### Measurements

Optimal systolic and diastolic BP was categorized respectively (≤120 mmHg and ≤ 80 mmHg). Pre-hypertension was also defined respectively for systolic (>120 mmHg and ≤ 139 mmHg) and diastolic BP (>80 mmHg and ≤ 89 mmHg). For hypertension, systolic BP was ≥ 140 mmHg, and diastolic BP was ≥ 90 mmHg.

BMI was calculated by dividing the weight (kg) of the respondent by the height in meters squared (m^2^). They were later classified as being overweight (BMI ≥25 kg/m^2^) and obese (BMI ≥30 kg/m^2^) according to the 2004 World Health Organization (WHO) cut-off points. Both BP and BMI were categorized to answer the first aim of the study, and for the second, they were considered as continuous variables. Leisure time PA is considered in this study. This is because it involves an activity which is planned, structured, and repetitively undertaken with the primary function of increasing physical fitness [30]. Therefore respondents were asked in the survey to describe the number of times they were involved in an activity in their free time in the past 7 days preceding the survey [31]. Subjects were categorized to have done no activity when all or most of their free time was spent doing things that involved little physical effort. For moderate activity, subjects who sometimes/often (1–4 times last week) did PA in their free time were termed as such whereas those who quite often/very often (5 or more times last week) did PA in their free time were considered to have done high levels of PA.

### Data analysis

The analysis was done using the IBM Statistical Package of Social Sciences (SPSS) version 19 (SPSS Inc. Chicago, USA). Percentages were used to determine the distribution of the youth by the variable of interest by sex. The association between PA, BMI and BP by sex were examined using bar graphs. Multiple linear regression analyses were also performed separately by sex in order to determine the factors influencing the associations of BMI and PA to systolic and diastolic BP; while adjusting for other related variables such as age, education, employment status, locality, smoking and alcohol consumption. P-value ≤0.05 were considered as statistically significant.

## Results

### Characteristics of the population

Table 1 shows the characteristics of the youth population under study by sex. There were 113 females and 88 males. About two-thirds (60.7%) of the youth were from Ussher Town. Almost half (42.8%) of the youth had completed basic education: there were more males than females who had completed basic education (50.0% vs. 37.2% respectively: p < 0.050). A little over a half (53.7%) of them were employed; a higher proportion of the males were employed compared to the females (65.9% vs. 44.2%; p < 0.050). We also present in Table 2, the levels of PA, BMI and BP. The youth were mostly of normal weight (67.2%). However, females had higher BMI than the males (23.6 kg/m^2^ and 21.7 kg/m^2^; p < 0.050 respectively) and a higher proportion of female youth were overweight or obese compared to male youth (17.7% vs 6.8% and 13.3% vs 2.3% respectively). About four-fifths (84.1%) were physically inactive: the male youth engaged in PA more than the female youth (15.9% and 4.4% respectively: p < 0.050). With respect to lifestyle, more males had either consumed alcohol or smoked tobacco compared to the females (p < 0.050). About a fifth (22.9%, former drinkers; and 28.9%, current drinkers) had either taken alcohol in the past or had been consuming alcohol in the past 30 days. More than one-tenth (14.4%) had ever smoked (Table 1).

**Table 1:** **Characteristics of the study population by sex**

		**SEX**		
	**All**	**Male**	**Female**	***P-value***
	**(n = 201)**	**(n = 88)**	**(n = 113)**	
**Age N (%)**				0.086
15-17	39 (19.4)	23 (2*6.1)*	*16 (14.2)*	
1*8*-20	67 (33.3)	25 (28.4)	42 (37.2)	
21-24	95 (47.3)	40 (45.5)	55 (48.7)	
*Mean*	*20.04*	*19.7*	*20.4*	
*Std. deviation*	*2.667*	*2.754*	*2.570*	
**Education N (%)**				0.002
No education	8 (4.0)	1 (1.1)	7 (6.2)	
Primary	46 (22.9)	11 (12.5)	35 (31.0)	
Middle/JHS	86 (42.8)	44 (50.0)	42 (37.2)	
Secondary +	61 (30.3)	32 (36.4)	29 (25.7)	
**Alcohol-use N (%)**				0.198
Never drinker	98 (48.8)	21 (23.9)	56 (49.6)	
Former drinker	46 (22.9)	25 (28.4)	21 (18.6)	
Current drinker	57 (28.9)	42 (42.7)	36 (31.9)	
**Smoked tobacco N (%)**				0.181
Never smoked	172 (85.6)	72 (81.8)	100 (88.5)	
Ever smoked	29 (14.4)	16 (18.2)	13 (11.5)	
**Locality N (%)**				0.579
Agbogbloshie	36 (17.9)	14 (15.9)	22 (19.5)	
James Town	43 (21.4)	17 (19.3)	26 (23.0)	
Ussher Town	122 (60.7)	57 (64.8)	65 (57.5)	
**Employment status N (%)**				0.002
Unemployed	93 (46.3)	30 (34.1)	63 (55.8)	
Employed	108 (53.7)	58 (65.9)	50 (44.2)

**Table 2:** **BMI, PA and BP levels of the study population by sex**

		**SEX**		
	**All**	**Male**	**Female**	***P-value***
	**(n = 201)**	**(n = 88)**	**(n = 113)**	
**Mean Height (cm)**	163.1	167.9	159.4	0.076
*Std. deviation*	*8.730*	*8.584*	*6.845*	
**Mean Weight (kg)**	60.5	61.1	60.0	0.080
*Std. deviation*	*12.576*	*10.876*	*13.823*	
**BMI (kg/m** ^**2**^ **)**	22.8	21.7	23.6	0.002
*Std. deviation*	*4.863*	*3.963*	*5.327*	
**Overweight N (%)**	26 (12.9)	6 (6.8)	20 (17.7)	
**Obese N (%)**	17 (8.5)	2 (2.3)	15 (13.3)	
**Systolic BP (mmHg)**	115.6	120.2	112.1	0.246
*Std. deviation*	*12.709*	*13.780*	*10.573*	
**Diastolic BP (mmHg)**	70.0	70.2	69.1	0.571
*Std. deviation*	*8.092*	*8.125*	*8.069*	
**Pre-hypertension N (%)**	65 (32.3)	37 (42.0)	28 (24.8)	0.009
**Hypertension N (%)**	8 (4)	6 (6.8)	2 (1.8)	0.069
**Physical activity N (%)**				0.000
No PA	169 (84.1)	62 (70.5)	107 (94.7)	
Moderate PA	19 (9.5)	14 (15.9)	5 (4.4)	
High PA	13 (6.5)	12 (13.6)	1 (0.9)	

### PA, BMI and BP levels

**Figure 1:**
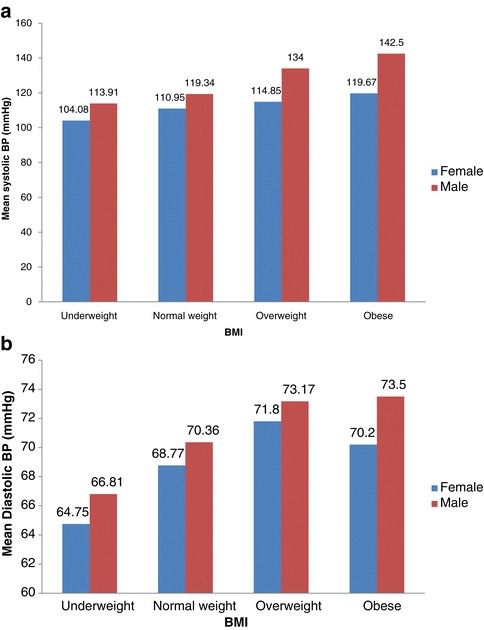
**Average Systolic and Diastolic BP and BMI of urban poor youth in Accra. (a)** Mean systolic BP by BMI and sex. Pearson correlation = 0.308; P-value = 0.000. **(b)** Mean diastolic BP by BMI and sex. Pearson correlation = 0.166; P-value = 0.018.

**Figure 2:**
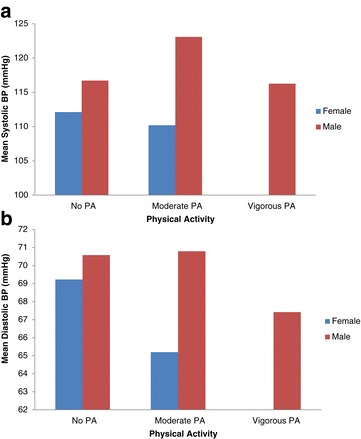
**Average Systolic and Diastolic BP and PA levels of urban poor youth in Accra. (a)** Average systolic BP and PA by sex. Pearson correlation = 0.066; P-value = 0.351. **(b)** Mean diastolic BP and PA by sex. Pearson correlation = −0.059; P-value = 0.402.

About one third (32.3%) of the youth had BP in the pre-hypertension category: a proportion of 42% of the male youth had pre-hypertension compared to 24.8% for females (p < 0.050). For hypertension, close to 7% and 2% were identified respectively for males and females: in total, 4% had hypertension. Average systolic and diastolic BP levels consistently increased with higher BMI; this was generally high for males compared to females – mean systolic BP for overweight [males: n = 6, SBP = 134 mmHg vrs females: n = 20, SBP = 114.9 mmHg] and mean diastolic BP for obese [males n = 2, DBP = 73.5 mmHg vrs females: n = 15, DBP = 70.2 mmHg] (Figure 1a & b). Similarly, higher levels of PA were associated with decreased levels of diastolic BP (Figure 2a and b).

**Figure 3:**
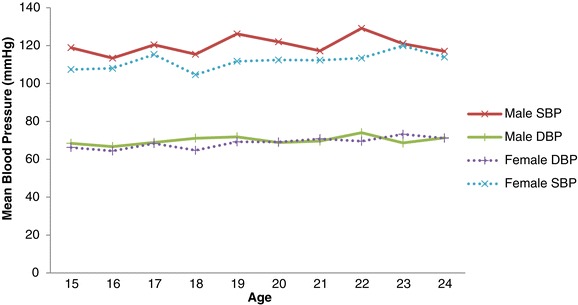
**Mean SBP and DBP by age and sex.** For Mean SBP: Pearson Correlation = 0.140; P-value = 0.047. For Mean DBP: Pearson Correlation = 0.200; P-value = 0.004.

Generally, male youth have high systolic and diastolic BP levels compared to females (p > 0.050). Both systolic and diastolic BP increased with age of youth and this was higher for males compared to the females, as Figure 3 depicts. Table 3 shows multiple linear regression analysis of factors associated with systolic and diastolic BP stratified by sex. The regressions showed that BMI was positively associated with only systolic BP (p < 0.050) for both male and female when confounding factors were accounted for. The influence of BMI on systolic BP was stronger for males compared to the females (β = 1.4 mmHg vs. β = 0.8 mmHg). Although PA was not significantly associated with systolic and diastolic BP in this study, moderate PA reduced BP level for both male and female youth, but high PA reduced BP for only males. For systolic BP, there was a decrease of −2.2 mmHg and −2.7 mmHg respectively for both moderately active male and female youth; however, for high PA female youth had an increase of 9.9 mmHg while the male youth a decrease of −3.8 mmHg.

**Table 3:** **Multiple linear regression of factors associated with systolic and diastolic blood pressure by sex**

	**Systolic blood pressure**						**Diastolic blood pressure**					
**Characteristic**	**Male**			**Female**			**Male**			**Female**		
	**β**	**SE**	**P**	**β**	**SE**	**P**	**β**	**SE**	**P**	**β**	**SE**	**P**
BMI	***1.382	0.368	0.000	***0.793	0.195	0.000	0.308	0.230	0.184	0.245	0.154	0.113
No PA (***REF***)	0			0			0			0		
Moderate PA	−2.157	3.396	0.527	−2.734	2.291	0.235	−3.215	2.117	0.133	−1.858	1.808	0.307
High PA	−3.787	4.939	0.446	9.933	10.036	0.325	−4.702	3.079	0.131	6.920	7.923	0.384
Age	0.108	0.625	0.863	0.342	0.443	0.441	0.606	0.389	0.124	0.409	0.350	0.245
Never smoker (***REF***)	0			0			0			0		
Smoker	−3.308	4.237	0.437	4.173	3.101	0.181	0.607	2.642	0.819	−0.445	2.448	0.856
Never drinker (***REF***)	0			0			0					
Former drinker	−2.315	3.582	0.520	1.665	2.658	0.532	1.591	2.233	0.478	2.621	2.098	0.214
Current drinker	0.487	3.967	0.903	1.366	2.317	0.557	0.490	2.473	0.844	0.875	1.829	0.633
Unemployed (***REF***)	0			0			0			0		
Employed	−3.749	3.516	0.290	−0.558	2.202	0.800	0.399	2.192	0.856	−1.243	1.738	0.476
No education (***REF***)	0			0			0			0		
Primary Educ.	−3.185	14.088	0.822	3.245	4.120	0.433	0.680	8.783	0.938	−1.756	3.252	0.590
Middle/JHS	1.837	13.763	0.894	3.664	4.077	0.371	4.723	8.580	0.584	0.276	3.218	0.932
Secondary +	3.385	13.802	0.807	7.165	4.353	0.103	6.863	8.604	0.428	2.051	3.436	0.552
Ussher Town (***REF***)	0			0			0			0		
Agbogbloshie	−2.079	4.495	0.645	2.749	2.539	0.282	3.457	2.802	0.221	**5.987	2.005	0.004
James Town	1.278	3.793	0.737	**5.120	2.441	0.039	0.893	2.365	0.707	**3.834	1.927	0.049
**Adjusted R** ^**2**^			**0.130**			**0.162**			**0.027**			**0.103**

***P < 0.0001; **P < 0.05; (***REF)*** **:** Reference category; **β:** beta coefficient; **SE:** Standard Error.

Among the confounders, locality was a significant predictor for both systolic and diastolic BP, and this was only significant for females. For systolic BP, females in James Town recorded an increase of 5.1 mmHg compared to those of Ussher Town. Similarly, the female youth from James Town had an increased diastolic BP of 3.8 mmHg compared to their counterparts from Ussher Town. Females from Agbogbloshie had a higher increase (β =5.9 mmHg) than those from Ussher Town.

## Discussion

### Key findings

Our findings suggest a high prevalence of pre-hypertension among the urban poor youth. Prevalence was higher in males than in females. Both systolic and diastolic BP increased with increasing levels of BMI; this was significant among males. About one tenth (11.5%) of female youth had ever smoked tobacco in the preceding 30 days.

### Discussion on the key findings

Our results add to the knowledge base on the dynamics of risk factors of HBP and mortality in Ghana. The male youth were found to have higher prevalence of pre-hypertension and hypertension than their female peers. This is consistent with studies of some developed countries, where cases of stroke and other CVDs are reported mostly among males due to their higher risk status for HBP compared to females [32-34]. Thus, males usually have higher morbidity and mortality of CVD and lower life expectancy than females [35]. The gender difference in this study is contrary to that of other rural and urban communities in Ghana, where girls aged 8–16 years had higher HBP compared to boys [5]. In a study among rural Ga communities, the mean SBP and DBP are higher in males than females aged 44 years and younger, but mean BPs were on the other hand higher in females than males 45 years and older [36]. This is consistent with a study in rural Northern region of Ghana among the adult population (18 to 65 years) where males aged 15–24 had higher SBP compared to their female counterparts who had higher DBP [37].

Primarily, the prevalence of overweight and obesity coupled with low PA among the urban poor youth may explain, at least in part, the HBP in these areas. A look at the influence of these two main variables indicated that an important variable of concern to HBP is BMI, which had a strong positive association. Similar positive associations were found in a study comparing Africans in Africa and in the African diaspora aged 35–64 years [38]. This supports recent evidence in the US where hypertension rates are increasing among overweight and obese young children aged 11 to 13 [39]. Therefore focusing on reducing or maintaining a balanced or ideal BMI is important in attaining good health. The influence of overweight and obesity in some urban areas, has led agencies like the WHO “to advocate using community (re)design as a tool to curb obesity” in some developed countries. Some of these advocated interventions include improvement of geographic availability of supermarkets in underserved areas, access to outdoor recreational areas and enhanced infrastructure to support walking [40].

This study revealed that females had higher levels of overweight and obesity than the males, yet there was a high average systolic and diastolic BP for each BMI level for the males, among whom BMI was significantly associated with systolic BP. Goon et al. [1] reported similar findings. In their study among children aged 7–13 years in rural South Africa, the incidence of elevated BP was high for boys compared to girls, although overweight occurred more in girls. Another study by Mkhonto et al. [41] in South Africa supports the association of BMI and BP; BMI and waist circumference were significant determinants of elevated systolic and diastolic BP for both male and female but only waist to hip ratio (WHR) was significant for males. However, females had high BP compared to the males. Several studies suggest that the mechanisms by which BMI influences hypertension are poorly understood and hence the sex differences are difficult to explain. It is unclear in this study the role of sex differences, although some factors have been thought to be influential. Of particular interest are modifiable determinants including physical inactivity, higher levels of alcohol intake and high levels of substance use; the females were more inactive, while males reported higher levels of alcohol intake and substance use. However, these factors were not significantly related with BP in this study.

Further in this study, PA showed no clear association with BP especially systolic BP. Mkhonto et al. [41] also found similar association in their studies. However, Luke et al. [17] advocate the health advantages of PA, and explain that it may solely not be the driving force behind the rise in obesity and other CVDs. In these urban poor communities, the youth were mostly inactive, since there are very few spaces for recreational activities. Hence, advocating for PA can help in maintaining a healthy body weight, thus reducing the risk of HBP [26] and further promote economic productivity since they form the majority part of the working force [42].

### Limitations

The study has some limitations. There is a potential bias in the self-reported participation of leisure PA. For instance, subjects’ recall bias effect may introduce some inconsistencies into the analysis. Also, there is a limitation with choosing only the frequency of leisure physical activity and not considering other forms of physical activity as well as their levels of intensity and duration, which could have better predicted the physical activity level of the respondents. We chose leisure activity as our operational concept because it involves a conscious decision and plan to engage in physical activity to increase physical fitness. However, it is important to consider that occupational activities in the communities involve manual work such as fishing, petty trading and artisan work, which some of the older respondents were engaged in. Such working activities involve energy expenditure which should be incorporated into a holistic measurement of physical activity. Finally the difficulty of establishing the cause and effect between physical activity and BMI does not allow for conclusive statements on the known benefits of physical activity. This may be attributed to the small sample size which could not allow for establishing the known associations between the variables. Despite these limitations, the findings in this study are consistent with findings from other studies and they provide insights that can inform interventions to lower HBP and cardiovascular disease (CVD) risk among youth in the research communities.

## Conclusion

Findings from this study indicate that the BMI of urban poor youth in some communities in Accra has a strong positive relationship with BP. The fact that youthful hypertension has the tendency of translating into adult hypertension suggests there is a need to prioritize research and interventions on HBP and its risk factors among adolescents and youth. The gendered differences on cardiovascular risk and health status require particular attention.

## Abbreviations

BP: Blood pressure
BMI: Body mass index
PA: Physical activity
HBP: High blood pressure
CVD: Cardiovascular disease
WHR: Waist to hip ratio
NCD: Non-communicable disease
LMIC: Low and middle-income countries
SSA: Sub-Saharan Africa
EA: Enumeration area
